# ‘ENVIRONMENTAL AKALISM’ AND THE WAR ON FILTH: THE PERSONIFICATION OF SANITATION IN URBAN NIGERIA

**DOI:** 10.1017/S0001972013000466

**Published:** 2013-11

**Authors:** John Manton

## Abstract

In Nigerian cities, as across much of Africa, sanitation practices at zone, ward and street levels inscribe – in patterns of circulation and interaction around waste – not only the hopes and fears of urban residents and managers, but also the aspirations and failures encoded in colonial and post-colonial national and regional histories. Adjusting to numerous challenges – the interplay of racist colonial zoning strategies, rapid post-colonial urban expansion, the withdrawal of public services amid the liberalization programmes of the 1980s, the increasingly abject character of the social contract, and the ongoing tenuousness of economic life and activity – urban environmental sanitation in Nigeria has long struggled to keep pace with the historical dynamics of the country's emergent metropolises. Following the activities of a cohort of inspectors and volunteers at the Ministry of Environment and Water Resources, Oyo State, this article examines the politics of performance and coercion surrounding the monthly observance of Environmental Sanitation Day in Ibadan amid the heightened political tensions of the electoral season in 2011.

## RÉSUMÉ

Dans les villes du Nigeria comme dans une grande partie de l'Afrique, les pratiques d'assainissement au niveau des zones, des quartiers et des rues inscrivent, dans des schémas de circulation et d'interaction autour des déchets, non seulement les espoirs et les craintes des résidents urbains et des gestionnaires, mais aussi les aspirations et les échecs encodés dans les histoires nationales et régionales post-coloniales. Confronté à de nombreuses difficultés comme l'interaction des stratégies de zonage coloniales racistes, la rapidité de l'expansion urbaine post-coloniale, le retrait des services publics qui a accompagné la vague de programmes de libéralisation des années 1980, le caractère de plus en plus abject du contrat social et la précarité persistante de la vie et de l'activité économiques, l'assainissement de l'environnement urbain au Nigeria peine depuis longtemps à suivre le rythme de la dynamique historique des métropoles émergentes du pays. En suivant les activités d'une cohorte d'inspecteurs et de bénévoles du ministère de l'Environnement et des ressources en eau de l’État d'Oyo, cet article examine la politique de la performance et de la coercition qui entoure la Journée de l'assainissement de l'environnement organisée tous les mois à Ibadan, sur fonds d'intensification des tensions politiques lors de la saison électorale en 2011.

Environmental sanitation connotes a series of social and technical practices converging on the circulation and management of waste from human activities. In the city, these practices are deeply intertwined with enterprises of public health and of urban planning, and with discourse on urban governance and rights in urban life. In Nigerian cities, as across much of Africa, sanitation practices at zone, ward and street levels inscribe – in patterns of circulation and interaction around waste – not only the hopes and fears of urban residents and managers, but also the aspirations and failures encoded in colonial and post-colonial national and regional histories. Adjusting to numerous challenges – the interplay of racist colonial zoning strategies, rapid post-colonial urban expansion, the withdrawal of public services amid the liberalization programmes of the 1980s, the often malign nature of urban and national politics, the increasingly abject character of the social contract, and the ongoing tenuousness of economic life and activity – the mismatched financial and human resources applied to urban environmental sanitation in Nigeria have long struggled to keep pace with the historical dynamics of its emergent metropolises.

The tasks facing policy makers and street labourers in environmental sanitation and waste management in the developing world are regularly conceived of in Augean terms; as evidence of systemic failures in urban governance, and of consistent threats to population health. In a recent review of the expanding literature on experiencing the African city in its rhythms and at its elusive and distributed margins, Jane Guyer notes a distinctive experiential category of ‘discontinuity’ in relation to infrastructural aspects of urban life as diverse as electricity supply, security and integrity of roads and drains, the constitution of public space, and the sufficiency of government and non-governmental planning, partnership and projects (Guyer 2011). The margins thus constituted are not simply geographical; they interrupt the conjoined enterprises of mapping and planning at their core and across their domain, infusing practices such as those relating to urban sanitation with a complex confection of necessity and futility, captured in every aspect of their performance, right down to the level of the street.

In the Nigerian case, approached here, these discontinuities are inscribed and read as evidence of a purposive collapsing of the domain of political action. Unfolding over the years of its oil boom, new forms of state action and lines of ministerial responsibility ‘emptied’ colonial governmentality, replacing mechanisms designed for rational administration and the maintenance of order with ‘tools of obstruction and interference’ (Apter 2005: 247). Andrew Apter queries the nature of a state that translated the euphoria of oil wealth into a carnival of plunder – harassing its population, spawning countless desperate strategies of subaltern dissimulation and accumulation, and presiding over environmental ruin – while its despotic acts of recrimination were crystallized in global consciousness by the execution in 1995 of Ken Saro-Wiwa and eight of his fellow Ogoni activists.

While Apter describes an instance of state fetishism become state vampirism under the reconfiguring pulse of oil – its flows, wealth and capture – Adebanwi and Obadare present us with the discarded corpse on which this vampire has fed, in a meditation on the abject character of a political and civic spectacle in which bodies are left to decompose by the roadside in proximity to flagship hotels. For these authors, the all-to-frequent impunity with which state organs disrupt livelihoods and exert force in the pursuit of nebulous and concealed aims is of a piece with the dereliction of civic and social space which marks popular disengagement from areas of state competence. The metonymic event at the heart of their reflections is a joint encounter with a corpse by the roadside while ascending the steep route to Ibadan's Premier Hotel. Able to deduce from experience the duration of the abandoned roadside afterlife of this ‘fellow citizen’, it is only on later reflection that the abject repercussions and resonances of this mute and accusing presence unfold (Adebanwi and Obadare 2010: 4–5). This signal of decomposition and degradation as an insistent existential murmur translates into a broader querying of political categories of state and citizenry, positing a malign core to government ritual, figuring the citizen as excremental cast-off, and focusing on insubordination as the primary means of enacting civic values in the face of state excess.

In this respect, the institution of Environmental Sanitation Day on the last Saturday of every month, one of urban Nigeria's more striking periodic rituals, exemplifies the spectacular simulation and unruly despair at the heart of civic life in Nigeria. It is a measure associated with historical and folk memory of the authoritarian and ostensibly anti-corruption military regime of 1983–5 led by Generals Mohammadu Buhari and Tunde Idiagbon. Conceived as part of the regime's War Against Indiscipline, Sanitation Day has been a monthly recurrence in Nigerian cities and towns throughout the last quarter century, despite the short tenure of the regime which imposed the measure. As currently practised, Sanitation Day consists of a period of restriction on movement (7–10 a.m., for example), a set of prescribed activities and routines, and a programme of monitoring and enforcement. The restrictive component of the day (or more properly, the morning) offers its own opportunities and risks; the prescribed activities and routines are both individual and corporate, comprising private and public sphere actors; and the programme of monitoring and enforcement links the restrictions and prescriptions, and ranges across the urban space.

And yet, in spite of the policed interactions between environmental inspection and communal and local responsibility for sanitation, the implementation of urban sanitation across the topographical and (imperfectly) class-stratified zones of the contemporary Nigerian city is tenaciously resistant to the mapping imaginary. It is to Ibadan, capital of Oyo State, that I turn in this presentation – the source of Adebanwi and Obadare's reflections on abjection; one of sub-Saharan Africa's largest cities by area, and one of Nigeria's most populous; and long perceived as especially subject to environmental mismanagement and catastrophe.

The 2011 electoral season saw the then Governor of Oyo State, Otunba (Dr) Christopher Adebayo Alao-Akala, fighting a desperate rearguard action to shore up the hegemony of the People's Democratic Party (PDP – the ruling party at national level) in the state. As part of the projection of social responsibility necessitated by electoral calculus, Akala trumpeted a spurious political philosophy headlined as ‘Environmental Akalism’. Political posters, Hilux trucks, and gleaming new white rubbish transporters propounded Environmental Akalism across Ibadan, without any contextualization or explication beyond the level of the slogan, and without any reference to the urban fabric or to the health and well-being of its inhabitants. Meanwhile, across the ‘interrupted landscapes’ (Guyer 2011: 478) of urban Ibadan, state-employed environmental health officers (EHOs) and voluntary marshals and cadets struggled to map and extend sanitary good practice along the street and into the compound and market, and to contain the debris of human activity.

This struggle has been more often tutelary than comprehensive; it performs and demonstrates at key times and places rather than enforcing, covering, and reaching across the entirety of the urban domain. In this, it adheres to and exemplifies some of the key features of a public realm in which power is articulated more as spectacle and interference than capacity, and experienced through practices of ordeal, evasion and abjection. In examining the foremost cosmetic performance in the sanitary calendar, and identifying some of the legacies and problems besetting both the spatial politics of environmental sanitation in Ibadan and the practice of policing, managing and collecting waste across the city, this article depicts aspects of a corrosive disenchantment which pervades political and civic action at the level of the city in Nigeria, and reflects the broader parameters of the traduced compact between Nigerians and the state.

## IBADAN, YORUBA METROPOLIS

Following Watson, Fourchard and Mabogunje, we can describe the site of Ibadan in relation to its hills, its streams, and their floodplains, as well as in relation to the historical constraints and opportunities that gave rise to concentrated settlement at its present-day site. As with many of the larger Yoruba cities of south-western Nigeria, Ibadan owes its origins to defensive responses to Fulani invasions from what is now northern Nigeria in the first half of the nineteenth century. By the last quarter of the nineteenth century, when British forces began to take a military and mercantile interest in the Yoruba interior, Ibadan had grown to be the largest and most militarily powerful of the Yoruba cities, and one of Africa's largest cities, with an estimated population of 200,000 by 1890 (Mabogunje 1962: 57). Watson, describing an important transitional time in the history of the city as it adjusted to British restrictions on traditional military and political activity, draws our attention to the evolution of a ward structure under powerful and carefully balanced local leadership, while Mabogunje outlines the impact of terrain on the development of urban residence (Watson 2000: 26–30; Mabogunje 1962: 59–60).

By the end of the British colonial era, Ibadan was already one of the most extensive cities in Africa, covering a land area of just over 100 square kilometres. Throughout the nineteenth century and the first half of the twentieth, Yoruba, other Nigerian and British occupants had built and moved into new residential areas on higher ground, leaving the lower reaches of the hills and the extensive flat flood plain (accounting for about two thirds of the city's land area) occupied by seasonal water, forest, and market gardening (Fourchard 2003: 3). Indeed, the identity of Ibadan as a colonial city was closely tied into its relation to a rich agricultural hinterland, and its position astride a number of significant trading routes and networks, not least the Lagos branch of the Nigerian railway. Ibadan was at the heart of Nigeria's lucrative cocoa belt, and derived wealth and a culture of urban display from this crucial colonial commodity. From 1948, Ibadan was at the centre of university education in Nigeria, with the foundation of University College (later the University of Ibadan); and from 1952, with the inauguration of regional government, it became the capital of the Western Region of Nigeria.

These developments led to a new influx of population from across Nigeria, bolstering the development of new African-occupied housing estates alongside the European Government Reserved Areas (GRAs), which also increasingly housed an African élite political corps alongside European administrators, merchants and scholars (Mabogunje 1962: 71–5). Industrial and infrastructural development added to the population and land pressures confronting the city, and, in the first decade after independence, much of the flood-prone land formerly used for forestry and farming began to be occupied on a more permanent basis. As in many African cities, the growth of urban population has been extremely rapid in post-independence Ibadan, from an estimated 600,000 in the early 1960s to somewhere between 2.8 and 6 million.^1^ This growth has outstripped the ability of the city to map and manage the infrastructural and ecological impact of new residents, and much housing is either inaccessible to large vehicles, or vulnerable to seasonal flooding that compounds the effect of poor waste disposal and drainage.

Since 1994 Ibadan has participated in the UN-HABITAT/UNEP Sustainable Cities Programme, with the ostensible aim of involving a broad range of stakeholders in all aspects of environmental management (UN-HABITAT and UNEP 2002: 29). It is difficult to determine, given the opacity of political activity, how seriously formulations around sustainability are intended to gain traction. The degree to which attempts to denote and consolidate sustainability as part and parcel of Ibadan's urban policy have remained hostage to political machinations at city, state and national level (see Omoleke 2004: 265–7) is not a primary consideration of this article. However, it is important to note that there exists a political and public vocabulary around issues of sustainability and rights in the clean city, and that this vocabulary is mobilized and invoked for a variety of purposes – which, unsurprisingly, do not always articulate well together.

## SANITARY CITIZENSHIP AND THE RETREAT FROM POLICY

SANITARIAN

Sure Safeguard for the Nation,

Against Environmental Challenges, through

Neatness and Cleanliness of Environment,

Immunization against Diseases and Improvement of Natural Environment,

Teaching all to Avoid Dirtiness,

Awareness Creation about Proper Health Habits,capable of,

Ridding the Society of Environmental Hazards, as

Inspector and Vanguard of Safe Living,

Arbiter and Symbol of Hygiene,an,

Arbiter and Symbol of Hygiene,an,

Non-Conformist to Unhygienic Conditions.

 While the practice and observance of Environmental Sanitation Day informs the development of sanitary and waste management in cities across Nigeria, and acts as a spur to regional and local policy, as of March 2011 there was no implemented national framework on sanitation for Nigeria, with responsibility for policy devolved to state level. As well as leading to differing times and dates across the country for the implementation of restrictions on movement, the resulting dependence on state ministries with environmental responsibility gave rise to an uncertain patchwork of waste management provision and policy, with an increasing reliance on private arrangements for the maintenance of drainage and the management and disposal of waste. The signature monthly practices of restriction and inspection had long been no more than etiolated reminders – merely cosmetic street-level signifiers – of the ‘frenzied sanitary activity’ of August 1985, when the fifth phase of Buhari and Idiagbon's War Against Indiscipline mobilized task forces, self-help groups, office and worker corps, and mobile sanitation courts to remove ‘permanent’ drain blockages and waste mountains, so renovating political and public discourse on sanitation in relation to human and urban health (Stock 1988: 19).

In merely renovating a politics of urban sanitation focused on local policing and individualized responsibility, with roots in the zonal and hierarchical mechanisms of colonial urban sanitation (Stock 1988: 21–3) – and inadequate to the huge growth experienced in the post-colonial city, not to mention the increase in post-consumer waste resulting from the Nigerian oil boom of the 1970s – the War Against Indiscipline never sought to engage with the exigencies of rapidly growing and industrializing cities. Cosmetic attention was paid to longstanding and highly visible waste disposal problems: neither water and sewage needs nor the threat of environmental pollution from industry were to be tackled in a concerted manner.

In all, this phase of the War Against Indiscipline, memorialized in minor ritual performances of state power and imprecations upon collective and public failures to tackle the ecological and environmental failure of Nigeria's cities, lasted less than one month: its pronouncement in Kano, northern Nigeria on 29 July 1985, accompanied by the offer of a one million naira prize (about £825,000 at 1985 rates) to be awarded to the cleanest capital, was followed on 27 August by the military coup of General Ibrahim Babangida. Babangida rowed back from the draconian nature of the War Against Indiscipline, revoked the prize fund, set in train a process of currency devaluation, and agreed a structural adjustment programme with the International Monetary Fund and the World Bank, eviscerating federal social provision and support for waste management and city planning.

Thus, while enforced and policed with rigour in its earliest military incarnation, the forms and outcomes of this ‘War’ have become somewhat sundered from the original sanitarian aspirations encoded in its draconian engagements over the intervening quarter century. For Idiagbon, every day was an Environmental Sanitation Day, presaging unheralded interference in the day-to-day activities of urban Nigerians. At the same time, some of the emphasis on the individual, on self-policing and neighbourhood surveillance, has persisted. This is reflected not only in popular banners, slogans and calendars as evidenced by the acrostic which opens this section of the article, but also in the voicing of behavioural expectations and the distribution of privatized means of environmental sanitation across the city.

The campus of the University of Ibadan, one of Nigeria's foremost universities and an exemplar of both the tutelary and the piecemeal nature of British colonial approaches to urban planning (Liscombe 2006), demonstrated the outlines and shortcomings of this model of urban sanitation, focused on surveillance, witnessed behaviour and performance, and cosmetic attention to the appearance of compounds and thoroughfares. While the university was not subject to municipal and state restrictions relating to Sanitation Day, its model of waste collection relied on an enforcing cadre of marshals whose theoretical street-level presence generated norms and expectations with regard to environmental conditions on campus, a routine of street sweeping tendered on a zonal basis to competing contractors, the privatized maintenance and collection of centrally located rubbish drums to which householders brought waste, and devolved responsibility for the cleanliness of individual housing compounds.^4^ Capital investment in the maintenance and construction of drainage was, as in the city at large, never more than sporadic and reactive.

Similar processes and practices existed in middle- and upper-middle-class areas of the city, with the upper reaches and gated areas of Bodija regularly mentioned as zones in which private sanitation and waste management arrangements prevailed.^5^ Consequently, the city was loosely stratified into those areas where private and community-funded arrangements earned a dispensation from the strictures of municipal surveillance (but not from restrictions on circulation); areas around markets and in lower-middle-class residential districts in which roads and drains were relatively well-defined and in which municipal trucks could circulate and project political power; and slum areas in which municipal environmental sanitation was little in evidence. The interpenetration of these areas across the city's topography, as described by Fourchard, and the reliance on a number of key routes for circulation across the city, rendered a policing structure based on a small series of checkpoints an effective mechanism for the projection of the particular politics of sanitation emerging from a combination of totemic performances of municipal environmental responsibility, and individuated and distributed spatial practices of surveillance and care.

## STAGING ENVIRONMENTAL SANITATION DAY

Dear Colleague, the bearer … is on special assignment with the Ministry … and he will be on the entourage of Honourable Commissioner for Environment for the monthly Environmental Sanitation Exercise between 6.00 a.m. – 9.00 a.m. on 26th March 2011. Kindly assist and allow him to pass through. Thanks.^6^

Of the practice of urban environmental sanitation in modern Nigeria, what is captured at the edge of a checkpoint in the centre of the government and business district, early on a Saturday morning? To address these questions in relation to my field encounter with the exercise of state power in the pursuit of urban sanitation, we need to consider the checkpoint in relation to its broader social, political, operational and performative contexts. We must ask: What kind of social event is a checkpoint? Around what sorts of (in)activity, sets of practices, and contextual markers does it cohere? And (in what ways) does it signify the successful or failed exercise of state power? In addressing these questions, we can unravel the strands of high politics and quotidian economic life refracted in the ethnographic encounter, and the extent to which the checkpoint weaves these and other strands of signification together – both loosely, as an arena in which a variety of personal practices cross and coexist, and more tightly, as a forum for the exercise of restrictive force and a totem of state prescription.

In relation to its performative nature, *waiting* is of course the modality of the checkpoint. All participants, willing or otherwise, face the prospect of being interrupted and detained, of engaging in waiting. The resultant *longueurs*, whether tense or bored, relaxed or irritable, are key to the performance and lived experience of the checkpoint. If we identify as an interface the transaction between authority – in the person of uniformed staff, questioner or armed individual – and the detainee or interrogatee, then the interface at the checkpoint counterpoints the threatening with the comic in varying proportions. Waiting and watching, the social world of the checkpoint enacts a theatre of domination and insubordination, but it is a theatre never devoid of ludic possibility. Indeed the Sanitation Day checkpoint I depict here, at the roadblock and its approaches, as well as behind the compound walls, offered ample scope for the performance of threat, banter, pleading, and the projection of roles of authority, citizenship, entreaty, submission and captivity. If we see this as a performance – more, I think, than just an anodyne exercise – this waiting, these *longueurs* punctuated by intermittent simulations of frenzy, generic in that they recur each month, constitute a commonly brokered trade-off between risk and opportunity that marks the psychic appeal and the grip of the checkpoint.

Just before dawn on Saturday 26 March 2011, I left the campus of the University of Ibadan with the IFRA driver, Mr Moses Ishola, and arrived at the Ibadan compound of the Ministry for Environment and Water Resources, Oyo State, where I was due to accompany the Commissioner – the Minister in charge, and a political appointee of the Oyo State Governor – and his environmental sanitation task force on their monthly inspection round of sanitation work in the city. My appointment was the culmination of four weeks of meetings, solicitations and deputations, convened with the aim of capturing the relation between state and private sector means of addressing Ibadan's grievous sanitation and waste disposal needs.

It seemed a particularly auspicious time to carry out these enquiries. Ibadan had a longstanding reputation for poor urban sanitation. Poorly planned post-colonial development had encroached on river banks and lands prone to seasonal floods, and the city had been subject to period outbreaks of cholera since the first major outbreak of the disease in 1971. Now, in early 2011, the improvement of environmental sanitation was not simply a stated priority of the Oyo State government, but a leading ideological plank in the re-election campaign of the incumbent Governor, Otunba (Dr) Christopher Adebayo Alao-Akala. His philosophy of ‘Environmental Akalism’ emblazoned trucks, skips and posters in the months leading up to the gubernatorial elections scheduled for April 2011. I looked forward to the opportunity to see this political philosophy in action.

On our way by 6 a.m., an hour before formal restrictions on movement began, we passed two night-time police checkpoints managing commercial traffic within the city and on the approaches to its markets. Mr Ishola drew attention to the gathering speeds of the vehicles aiming to reach their destinations before restrictions began. Between 6 and 6.30 a.m., members of the environmental sanitation task force began to meet and gather at the compound, and I presented my letter of authorization to signal my presence, before passing the letter to the IFRA driver to ensure his safe passage back to the university.

The task force was made up of white-uniformed environmental health officers (EHOs), and a number of vehicles were parked in the compound forecourt, with one especially conspicuous white Hilux truck bearing the prominent legends ‘Keep Oyo State beautiful and clean – Akala’, and ‘Environmental Akalism’ on its side and rear. Looming over the compound was a political billboard supporting Alao-Akala, declaring ‘Renewed passion: higher goals’. Outside the compound, across a stretch of dual carriageway which cut from the university, through Bodija, and down towards the Oyo State Secretariat and University College Hospital, a roadblock was being set up. The apparatus consisted of four spiked strips, two on each side of the highway, with six triangular warning signs mounted on defunct truck wheels, amidst and at either edge of each spiked strip set-up. The roadblock was unusual in cutting off all passage, as opposed to the usual policing practice of restricting passage to a pinch point. Prior to the imposition of full restriction on movement, the corps staffing the roadblock would lift one spiked strip and indicate a through route to approaching buses, cars, bikes and trucks.

The approach and exit from each blocking point was staffed by a lightly armed volunteer corps made up of three distinct uniformed groups: the Scouts, in green shirts with berets and a variety of neckties; the Royal Rangers, wearing light tan shirts and trousers; and the Man O’ War corps, wearing a dark brown uniform and wide-brimmed brown hat. Most carried no weapons, while some bore a short coiled-rope whip. Upon enquiry, a member of the Man O’ War corps described the group as ‘a voluntary organization that helps the government’, with no express indication of what other functions they might be mobilized to discharge. Uniformed, unarmed, and young, none of the groups manning the checkpoints corresponded to the image – of unstable and unpredictable gangs of under-employed youth beholden to politicians – commonly deployed in analyses of Nigerian political life since 1998. All three bodies had a national presence and a strong voluntary ethos, but it was impossible to ascertain, in the brief time I spent at the compound, the background, hierarchy and opportunities made available to members of any of these groups.

The simulacrum of responsible governance enacted at and around the Sanitation Day checkpoint was compounded in its unreality by the attendance of a band of young journalists from the various news organs in the city. Assembled and addressed throughout as a group, their first performance consisted of an informal mimicry of the roadblock corps, who had begun to direct infringing motorists and travellers towards the compound interior. ‘Enter, enter’, the journalists directed, before taking seats on a low wall outside the compound gates to share stories and notes. Behind them, one more senior journalist with a cameraman in tow interviewed the Chief EHO on the importance of Environmental Sanitation Day in the communal life and public presentation of the city. Later in the morning, the group of journalists was summoned inside the compound to listen to a prepared statement on the work of the Ministry, its task force, and its corps of EHOs.

The final and key component in the social universe of the Sanitation Day checkpoint was the magistrate's court. The EHO who had briefed me on the monitoring duties of the task force had also informed me that across the 5 LGAs of the city, containing 20–25 enforcement points in total, there were also 6 or 7 magistrates sitting, in order to punish the infringement of Sanitation Day regulations, whether by travelling or by failing to maintain a clean streetscape. One such minimally convened court was sitting in the porch of the Ministry building. Two tables were placed side by side, with the magistrate seated at the one closest to the security doors of the Ministry, and her two clerks at the other. An officer in a black Nigerian Police Force uniform stood behind the tables and their occupants, and beside her stood an EHO. Two queues formed initially, with a man with sunglasses in a brown uniform marshalling from the rear, before it was decided to contain the crowds of captive travellers in the foyer behind the security gates of the Ministry, and release them one by one to be confronted with their dereliction in respect of the sanitary responsibilities of the citizen, and to plead for clemency or the application of a special exception on the grounds of one or other specific rationale behind their travel. In all cases I witnessed – I was not allowed to attend too closely, nor to photograph proceedings – a substantial on-the-spot fine was administered and collected. Vehicles such as motorbikes, buses and trucks (one carrying an enormous and well-worn public address system) were directed into the compound and either parked for the duration of the exercise, or impounded until payment of fines was secured.

## THE CHECKPOINT AS PERFORMANCE

It is when we see the state improving on what we are leaving behind that we shall feel happy that our efforts have not been in vain. We are conscious of the fact that government is a continuum, we cannot do all, but we can walk tall and beat our chest that we have done our best in the last four years.

Outgoing Commissioner for Environment and Water Resources in Oyo State, Majekodunmi Aborode (Ogunsola 2011)

Significantly, the checkpoint here comprised more than just the roadblock. Set on a key business and trading route, and rendered difficult to evade by the tortured urban geography of the city, the roadblock – and the restrictive activities that it enabled – relied on an enclosed hinterland in which vehicles could be impounded, the movement of detainees restricted, and the offices of the magistrate afforded their due respect and privacy. The extensive and functionally differentiated nature of the checkpoint acted as a guarantee against the more egregious and tyrannical excesses, and the more opaque aspects of performance and permission associated with roadblocks, as described by authors such as Johan Pottier (Pottier 2006: 165–75).

As the corps readied themselves for the 7 a.m. deadline, a number of waste disposal trucks were indicated to me by one of the EHOs. Some were old and lumbering, but one stood out in its gleaming magnificence: a brand-new white truck with state-of-the-art compacting equipment and a high-visibility comfortable cabin. Painted on the side was another variant on one of Alao-Akala's signature statements: ‘Keep Oyo State clean and beautiful … : Akala’, and underneath, in cursive script, ‘*Environmental Akalism*’. I noted that a number of the EHOs were wearing white t-shirts in place of the regular white shirt uniform. On the back of one type of t-shirt was printed ‘“Many Species, One Planet, One Future” – Oyo State Ministry of Environment and Water Resources 2010 World Environmental Day’. A senior EHO briefed me on the timetable for the morning, indicating that the task force of EHOs in the compound were awaiting the Commissioner in order to proceed with the inspection that formed the central component of the monthly environmental sanitation exercise. Once he had arrived, the entourage would proceed to monitor the state waste disposal trucks to ensure that they were carrying out their duties, and inspect surrounding neighbourhoods to see that drains and roads in front of residences were being cleared by the residents.

The relationship of the monitoring duties of the task force to the totality of sanitation work in the city was thus starkly and honestly delineated for me. It was not the responsibility of the state to ensure monitoring of the privately arranged waste collection that many middle- and lower-middle-class neighbourhoods funded, nor, it seemed, to police the ultimate site of disposal of waste. Mr Ishola later suggested to me that Alao-Akala's signature white trucks were only in evidence in the wealthier areas of the city, and indeed restrictions on vehicular access in many of the old areas of the city, which coincided with some of the poorest and many of the most poorly-kept areas, would tend to corroborate this impression (Fourchard 2003: 6). Further, the task force's monitoring duties covered only the five LGAs of the city proper, and didn't extend to the entire metropolitan area. In each of these LGAs, embracing over 100 square kilometres, there were four or five ‘enforcement points’, mirroring the checkpoint at which the EHOs and I awaited the Commissioner, convened with the intention of forcing people to remain at home, where they might be stimulated or encouraged to clean compounds and to take responsibility for their local streetscape.

The decals of Environmental Akalism were everywhere in evidence around the compound of the Ministry, commissioned – and presumably ready to be decommissioned – on reputational and electoral whim. While some of the EHOs wore full uniforms with white short-sleeve shirts, epaulettes, and olive berets, others wore white t-shirts sporting slogans referring to environmental achievements under the stewardship of Chief Adebayo Alao-Akala. His signature quote, a variant on ‘Keep Oyo State clean and beautiful’, adorned t-shirts, Hilux trucks and brand new rubbish transporters criss-crossing the metropolis, and most spectacularly in evidence on this particular morning of the monthly sanitary-political calendar.

The proximate context, then, for the work of the checkpoint at which I found myself was the ongoing bluster surrounding environmental sanitation at a state level, where notions of the clean city and good environmental stewardship were hailed by the Governor, Alao-Akala. What was striking about Environmental Akalism was that, despite persistent infrastructural problems with drainage, road construction, market hygiene and waste management, it was publicly feted as a viable and potent political philosophy throughout the latter part of his governorship. Its purpose was not only to signify the strides made by the PDP administration of Oyo State in improving the surroundings of all citizens of the state, but to emblazon them in the public space of the city and its streets, by means of waste-disposal trucks, conferences, celebrations of global environmental events, and a variety of t-shirts, decals and slogans that conjoined sanitary pabulum and grandiose political projections.

In total, the Sanitation Day checkpoint consisted of a roadblock mounted and manned outside the gates of the Oyo State Ministry of Environment and Water Resources compound; four separate corps of EHOs (comprising the task force) and voluntary groups of mostly young men, all uniformed; a parking area, which doubled as a vehicle pound; a group of attending journalists; and an outdoor magistrate's court complete with police support. While awaiting the arrival of the commissioner, in his galvanizing role, I left the compound to observe the labours and transactions involved in enforcing and negotiating the roadblock itself. As the 7 a.m. cut-off point drew near, vehicles approached and passed the compound at ever-increasing speeds until, just prior to the turning of the hour, the spike strips were moved into place. From then on, volunteers bunched in twos and threes, and lined the approach to the strips in order to slow vehicles on their approach.

Drivers were instantly confronted and held to account for their presence on the roads, and, as queues of vehicles began to develop, oncoming cars and vans could be seen to turn and retreat in the near distance. Drivers and volunteer corps began to argue in heightened tones, as drivers disputed the reasons for their presence on the roads. Hospital and health workers, travelling in cars or on the pillions of motorbikes, were waved through once their identity as key workers could be established, and motorbikes with non-key staff often took advantage of developing arguments, or temporary breaches to let permitted vehicles through, to race around the gathered corps and speed off into the distance. This gambit was not always successful, as volunteers crowded round bikes and attempted to remove ignition keys or steer the bike towards the drain, using rope whips to dissuade pleading or flight, and to direct rider and passenger towards the compound where they would face the magistrate's court.

One driver attempted to run through the spike strip, only to be instantly immobilized by punctures to both front tires and a failure to shake off the strip, which had to be removed manually. This driver, too, was escorted into the compound. Later, as traffic began to die down and the volunteers became less energetic in their pursuit of infringing drivers, a bus laden with goods and full of traders, coming from the Secretariat in the direction of Bodija market, stopped at the far side of the road about 20 metres short of the checkpoint. Each occupant jumped from the bus and ran along the inner edge of the drain alongside the road, some bearing plastic bags or sheets loaded with goods. A brief chase was given, but none of the occupants were brought back to face the magistrate. The ability of certain traders and motorcyclists to evade the attentions of the youth corps demonstrated a recognized limit to the power and threat which could (or would) be mobilized by the state in pursuit of sanitary aims.

By 8.15 a.m., it became clear that the Commissioner was not going to attend on that morning. Groups of volunteers had begun to chase one another, giving way to playful shoves and idle chat along the central reservation. By this time, a pair of women with brooms could be seen approaching from the direction of the Secretariat. Each picked waste from the roadside, and swept dust off the road towards the central reservation and the drains on either side of the road. Unheeded, they eventually passed the by-now inattentive volunteers and the EHOs who had resigned themselves to awaiting the end of the time allotted for the exercise. The two women went on sweeping, as they did along the dual carriageway six days a week, not raising heads to engage with any aspect of what passed for an environmental sanitation exercise at the compound of the Ministry.

The magistrate continued to process and discharge detainees, who subsequently gathered outside the gates of the compound to await the resumption of mass transport. At bus stops equidistant on either side of the roadblock, vehicles could be seen massing from about 8.45 a.m., and, on the lifting of the roadblock, both sides of the carriageway almost instantly filled with laden buses, cars and trucks, as the economic life of the city resumed without a backward glance. The roadblock was quickly tidied away, volunteers and EHOs retreated inside the compound, and I was told that the exercise was over. I was free to leave, and the city was once again free to go about its business unsupervised and unmonitored.

It turned out that the Commissioner had been summoned by more pressing political duties, in a vain attempt to shore up Alao-Akala's foundering re-election campaign. All the same, the morning and the exercise had seemed to labour under the sign of dysfunction, or, at the very least, of function deferred. For all the effective discharge of quasi-state functions visibly associated with the checkpoint and its satellites – the work of the magistrate, the TV and print journalists, the EHOs sensitizing the population, and the volunteers maintaining quiet streets in the city – there was a clear disjuncture between the political performance and public projection of sanitation as a responsibility of the citizen fulfilled in concert with the state, and the construction of a punitive rent-seeking apparatus almost wholly disjoint from the environmental health of the city.

Environmental Sanitation Day had become a hostage to the political calendar. The 2011 election season, which saw national legislative, presidential, and state gubernatorial campaigns and elections take place in March and April, gave rise to the cancellation in Lagos State of sanitation exercises and restrictions on movement scheduled for that morning of Saturday 26 March. The same date on which I sought to examine the conduct of sanitation work in Ibadan, Oyo State, saw politicians and high office holders across the country absent themselves from the levers of government, so as to apply themselves to securing the perpetuation or succession of their political dynasties.

Throughout this exercise, EHOs were rendered both highly visible in their expectant gathering and grouping across the extensive space commandeered by the checkpoint (the roadblock and its adjunct services), and frustratingly immobile by the failure in duty and performance of their politically appointed superior. Their superfluity left bare the apparent rent-seeking nature of the checkpoint. Even so, the transactions surrounding the extraction of rents were not flattened into a simple exercise of force, a straightforward and violent expropriation. While the particular circumstances of the day brought to the fore the extent to which the cosmetic and performative have substituted for adequacy of coverage in the pursuit of urban sanitation, the process by which money changes hands was conducted according to rituals that assumed and projected a performance of probity.

The drawn-out interactions that comprised the capture and processing of detained motorists and passengers were not conducted in a despotic fashion. Indeed, the compact between authority and detainee demonstrated an assent to the conduct of unarmed uniformed youth that seemed as much dependent on one's attachment to one's vehicle, or on the weight and portability of one's goods, as on any overt threat of violence. Those who could carry and run, or drop and scatter, did so. Separated from their vehicles with the tacit assurance that neither goods nor vehicle would be appropriated without due cause (such as failure to pay the fine), detainees were marshalled into the presence of the magistrate and waited their turn.

Thus, the summons to activity – the gathering and scattering – that accompanied the advent of a minibus full of traders encapsulated registers of duty, rent seeking, livelihood management, political and economic *interference*, and the exercise of state and para-state power. The passage through the rapidly convened and yet carefully differentiated apparatus of detention and punishment was punctuated with entreaties and outbursts that seemed to indicate a chaotic and arbitrary nature to proceedings, but the clearly articulated, relatively transparent, and well-ordered nature of the discharge of punitive functions distinguished this particular checkpoint from many others at roadsides up and down the country, equally official in appearance but far less deeply entrenched in the practice of statecraft, and far more dangerous, threatening and arbitrary in their functioning.

This strong functional differentiation, the sensibility on the part of all of those working at the checkpoint that they were engaged in the work of government, and the reference to broader social action and responsibility attest to a certain degree of assent accorded among the population at large to the processes surrounding environmental sanitation, however mired in a failed and traduced model of political performance, and a subversion of the practice of public health. Taking the city as a whole, the checkpoint was metonymic of the performance of environmental sanitation across the urban space, conjoining stern supervision at a small number of checkpoints – five or six in total, one EHO indicated to me – with widespread evasion and minimal evidence of care for the urban fabric.

## CONCLUDING REMARKS

You need to see the way people of this area often throw garbages into gutter anytime rain is falling. I don't know why people [take] delight in this dirty habit that could trigger flood and outbreak of diseases. Government needs to deploy environmental officers to this area to curb this menace.

Alhaji Ogunlade Mudashiru, resident of Saka Pena, Ibadan (Adeniyi 2011)

In the context of sanitation day in Ibadan, events relating to Environmental Sanitation Day increasingly took place under the sign, as it were, of Environmental Akalism, in relation to its signifiers, both missing (as in the case of the Commissioner on secondment to Akala's soon-to-fail gubernatorial campaign) and manifest (in all the magistracy of the *al fresco* court). Thus, the checkpoint as social event coheres around a set of practices related to social responsibility even if none of the practices we would normally associate with the discharge of such responsibilities are explicitly performed at the site of the roadblock. In this respect, ‘Environmental Akalism’ as a performance, and a political philosophy, exemplifies in an especially distilled form the analytical independence of ‘environmental sanitation’ in Nigeria from urban waste and environmental management, and urban planning and mapping as it is actually enacted.

In its variety of uniforms (and their occasional absence), in the attendance of a coterie of journalists, closed in on themselves and barely interacting with the spectacle at large, and in its hastily arranged and yet clearly delineated functional and material dispositions, the sanitation day checkpoint bore a mystifying relation to the quotidian practices of urban sanitation and waste disposal. Its mystification lay in the crucial and yet tangential relation between the checkpoint as a social event and political spectacle, and the set of practices related to public health and social responsibility around which it ostensibly cohered. There is a significant disjuncture between the disciplinary and rent-seeking functions served by this particular genre of checkpoint, and the functioning of the ideal city with which it bears a metonymic relation.

The projection of power at the heart of the punitive functioning of the checkpoint signified both the repressive remnants of the War Against Indiscipline which lay at the roots of the performance of urban environmental sanitation as currently constituted in Nigeria, and the political reflex which sought to impel the citizen to reproduce surveillance and cleanliness as statecraft across the extent of the city. At once a theatrical venality, and a symbol of the excess of the state theorized by Adebanwi and Obadare, which eviscerates civic discourse and perpetrates the abjection rampant in contemporary conceptions of Nigeria, Environmental Akalism and its public projection reproduce a disjuncture between public expectations regarding stated policy, and the experience of (non-)implementation and the abandonment of the citizen as a focus of policy.

This complex of policed rights to circulate in the city, taken together with the institutionalized neglect of the urban fabric, links the manipulation of space in the pursuit of environmental sanitation in Ibadan to the failure and dereliction of politics as a means to address the needs of Nigeria's urban population. The (incomplete) street-level performance to which I was witness – originating in my interviews in the offices of the university, further engaging and entreating the ministerial bureaucracy, and finally attending and awaiting the discharge of surveillance and sanitation duties – was both grafted onto a poorly extended and mapped, dispersed, and stratified apparatus for environmental management, and marshalled as a totem of political responsibility and showmanship. The performance, together with the range and extent of municipal functions that it indexed, was a crucial component of the fabric of urban experience, life and politics in Ibadan, and a signal of the bankruptcy of urban politics in terms of rhetoric and repertoire.

Recent events have highlighted the historical shortcomings in urban spatial management and sanitation and waste disposal in Ibadan, Oyo State, Nigeria, as heavy rains in late August 2011 led to unusually severe flooding. The flooding and its mismanagement led to the deaths of over 100 people, and compounded the almost annual risk of recurrence of cholera in the city's slums and hospitals, leading to an outbreak of the disease in September. The university's drainage system, in common with many drains in high- and low-lying areas across the city, was overwhelmed. The new Governor of Oyo State, Abiola Ajimobi, has borrowed from the despotic repertoire of previous administrations, issuing fiats aimed at destroying the hitherto tolerated right of residency and construction in and around the city's main avenues of drainage and water supply, and forcing markets to close on Thursday mornings for a new weekly sanitation exercise. Alao-Akala's signature white rubbish trucks now bear the cursive legend ‘Environmental’, with ‘Akalism’ excised. It remains to be seen whether the underlying strategies for environmental management can shift from a focus on draconian rhetoric and personal responsibility, to one of investment in urban infrastructure, widely dispersed street-level waste management machinery and personnel, and a refurbishing of civic responsibility across the variegated landscape of Ibadan's neighbourhoods and arteries.

## Footnotes

1 The figure of 2.837 million is cited as a 2010 estimate at <http://esa.un.org/wup2009/unup/index.asp>. A variety of upward revisions exist but all estimates need to be qualified with reference to the political sensitivity of census and population figures in Nigeria, where allocation of revenue at state and local government level is determined with respect to population (see Fourchard 2003: 3).

4 Interview with Mr Charles Ojo, UI Estates Office, and Martin Mbella, 14 March 2011.

5 Interviews with Ojo, Adams and Mbella, March 2011.

6 Letter dated 25 March 2011 from R. E. Oyemde, Chief Environmental Health Officer, to whom it may concern, authorizing my free passage on the morning of the Environmental Sanitation Day exercise, Oyo State.

## References

[ref1] Adebanwi W. and E. Obadare (2010) ‘Introduction: excess and abjection in the study of the African State’ in W. Adebanwi and E. Obadare (eds), Encountering the Nigerian State. New York NY: Palgrave.

[ref2] Adeniyi S. (2011) ‘Insufficient refuse collection facilities responsible for flooding in Ibadan – Residents’, *Nigerian Tribune* (Ibadan), 24 June, <http://tribune.com.ng/index.php/community-news/24005-insufficient-refuse-collection-facilities-responsible-for-flooding-in-ibadan-residents>, accessed 14 July 2013.

[ref3] Apter A. (2005) The Pan-African Nation: oil and the spectacle of culture in Nigeria. Chicago IL: University of Chicago Press.

[ref4] Fourchard L. (2003) ‘The case of Ibadan, Nigeria’ in Understanding Slums: case studies for the Global Report 2003. London: Development Planning Unit, UCL and UN-Habitat, <http://www.ucl.ac.uk/dpu-projects/Global_Report/pdfs/Ibadan.pdf>, accessed 14 July 2013.

[ref5] Guyer J. I. (2011) ‘Describing urban “no man's land” in Africa’, Africa 81 (3): 474–92.

[ref6] Liscombe R. (2006) ‘Modernism in late imperial British West Africa: the work of Maxwell Fry and Jane Drew, 1946–56’, Journal of the Society of Architectural Historians 65 (2): 188–215.

[ref7] Mabogunje A. (1962) ‘The growth of residential districts in Ibadan’, Geographical Review 52 (1): 56–77.

[ref8] Ogunsola O. (2011) ‘Alao-Akala's success on environment will haunt Ajimobi – Commissioner’, *Daily Independent* (Lagos), 27 May, <http://allafrica.com/stories/201105300789.html>, accessed 14 July 2013.

[ref9] Omoleke I. I. (2004) ‘Management of environmental pollution in Ibadan, an African city: the challenges of health hazard facing government and the people’, Journal of Human Ecology 15 (4): 265–75.

[ref10] Pottier J. (2006) ‘Roadblock ethnography: negotiating humanitarian access in Ituri, eastern DR Congo, 1999–2004’, Africa 76 (2): 151–79.

[ref11] Stock R. (1988) ‘Environmental sanitation in Nigeria: colonial and contemporary’, Review of African Political Economy 42: 19–31.

[ref12] UN-HABITAT and UNEP, Sustainable Cities Programme (2002) 1990–2000: a decade of United Nations support for broad-based participatory management of urban development. Nairobi: UN-HABITAT.

[ref13] Watson R. (2000) ‘Murder and the political body in early colonial Ibadan’, Africa 70 (1): 25–48.

